# Sustainable biopolymer soil stabilisation: the effect of microscale chemical characteristics on macroscale mechanical properties

**DOI:** 10.1007/s11440-022-01732-0

**Published:** 2022-12-11

**Authors:** Samuel J. Armistead, Colin C. Smith, Sarah S. Staniland

**Affiliations:** 1 Department of Chemistry, The University of Sheffield, Dainton Building, Brook Hill, Sheffield, S3 7HF UK; 2 Department of Civil and Structural Engineering, The University of Sheffield, Sir Frederick Mappin Building, Sheffield, S1 3JD UK

**Keywords:** Biopolymer, Cross-scale, Design principles, Galactomannan, Micro to macro, Mine tailings, Stabilisation, Sustainable geotechnics

## Abstract

Sustainable biopolymer additives offer a promising soil stabilisation methodology, with a strong potential to be tuned to soil’s specific nature, allowing the tailoring of mechanical properties for a range of geotechnical applications. However, the biopolymer chemical characteristics driving soil mechanical property modifications have yet to be fully established. Within this study we employ a cross-scale approach, utilising the differing galactose:mannose (G:M) ratios of various Galactomannan biopolymers (Guar Gum G:M 1:2, Locust Bean Gum G:M 1:4, Cassia Gum G:M 1:5) to investigate the effect of microscale chemical functionality upon macroscale soil mechanical properties. Molecular weight effects are also investigated, utilising Carboxy Methyl Cellulose (CMC). Soil systems comprising of SiO_2_ (100%) (SiO_2_) and a Mine Tailing (MT) exemplar composed of SiO_2_ (90%) + Fe_2_O_3_ (10%) (SiO_2_ + Fe) are investigated. The critical importance of biopolymer additive chemical functionality for the resultant soil mechanical properties, is demonstrated._._For Galactomannan G:M 1:5 stabilised soils the ‘high-affinity, high-strength’, mannose-Fe interactions at the microscale (confirmed by mineral binding characterisation) are attributed to the 297% increase in the SiO_2_ + Fe systems Unconfined Compressive Strength (UCS), relative to SiO_2_ only. Conversely for SiO_2_ Galactomannan-stabilised soils, when increasing the G:M ratio from 1:2 to 1:5, a 85% reduction in UCS is observed, attributed to mannose’s inability to interact with SiO_2_. UCS variations of up to a factor of 12 were observed across the biopolymer–soil mixes studied, in line with theoretically and experimentally expected values, due to the differences in the G:M ratios. The limited impact of molecular weight upon soil strength properties is also shown in CMC-stabilised soils. When considering a soil’s stiffness and energy absorbance, the importance of biopolymer–biopolymer interaction *strength* and *quantity* is discussed, further deciphering biopolymer characteristics driving soil property modifications. This study highlights the importance of biopolymer chemistry for biopolymer stabilisation studies, illustrating the use of simple low-cost, accessible chemistry-based instrumental tools and outlining key design principles for the tailoring of biopolymer–soil composites for specific geotechnical applications.

**Graphical abstract:**

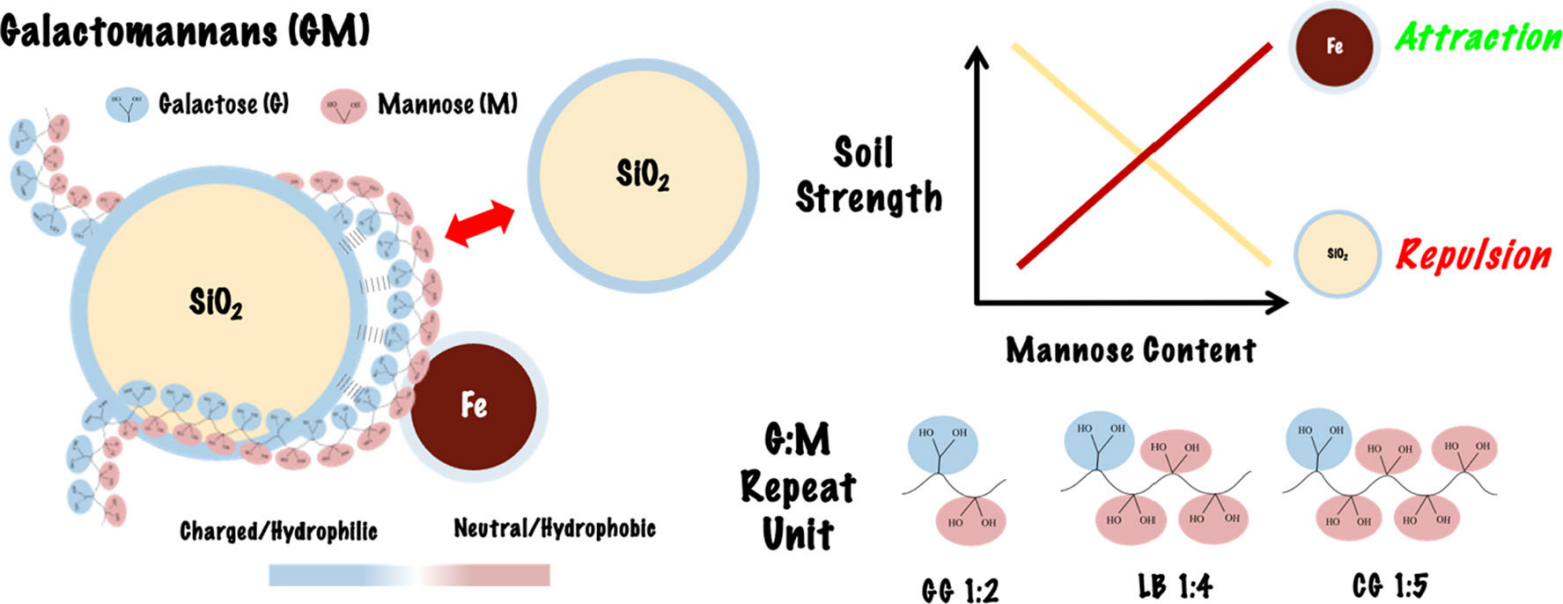

**Supplementary Information:**

The online version contains supplementary material available at 10.1007/s11440-022-01732-0.

## Introduction

Stabilisation/solidification of soils is desirable for a range of applications such as improving foundation stability [25], mine tailing stabilisation [40], the construction of geotechnical structures [48], the production of building materials [17] and the prevention of soil erosion [46]. Typically, cement-based additives are used to improve soil characteristics [7]. However, cement exhibits a host of environmental damaging characteristics: critically it being a major contributor to global carbon dioxide emissions (1 tonne of cement = 1 tonne of CO_2_) [3].^,^ Due to the current climate crisis, a shift towards sustainable, low carbon, geotechnical solutions is crucial.

Chemical [60], physical [1, 10], electrical [30] and biological [56]-based soil strength improvement methodologies have been developed to provide low carbon cement alternatives. Chemical methodologies, such as alternative inorganic cementation (e.g. lime, fly ash, ground blast furnace slag, silica fume) and polymer-based additives (e.g. bitumen, synthetic polymers, epoxy resins), have been employed; however, their derivation from industrial and petroleum sources, along with their potential environmental impacts, is of major concern [22, 45, 54]. Physical stabilisation, such as the use of mechanical action (e.g. compaction, vibration, soil mixing) and reinforcements (e.g. geosynthetics, fibres), has also been applied; however, due to the lack of chemical bonding, they typically exhibit lower bearing capacities and durability concerns [6, 28, 58]. Biological-based microbial/enzyme-induced calcite precipitation has gained research interest due to their sustainable qualities [53]. Their bio-catalytic nature however results in a sensitivity to certain soil types and conditions [57]. The field would benefit from a stabilisation method which can be easily tuned to soils heterogeneous nature, whilst exhibiting the inherent sustainable characteristics required to address the climate crisis.

Biological derived polymers (biopolymers) are attracting increased attention due to their desirable qualities, such as renewable sources, low carbon production, low toxicity, local availability and their increasing economic viability [13]. Biopolymers further offer a vast catalogue of chemical functionalities, due to their synthesis within nature to fulfil many different biological functions (energy storage, structural support, gelling agents) [8, 59]. Their ex situ production through exo-cultivation or chemical extractions, also offers a high control over production, preparation and addition methodologies [16]. Further inspiration for their use has also come from the emerging importance of biopolymers within strong natural bio-mineral composite materials such as bone, teeth and shells [37]. A number of reviewers have highlighted their significant potential within both geotechnical and construction soil-based applications [24, 36, 50]. Previous studies researching the use of biopolymer additives have found superior soil strength improvements relative to cement-stabilised systems [15].

In previous work we have introduced a ‘micro to macro’, Membrane Enabled Bio-mineral Affinity Screen (MEBAS)—Mineral Binding Characterisation (MBC)—Geotechnical Verification (GV) methodological pipeline, capable of identifying high-strength biopolymer–soil composites with an approximately 50-fold increase in the rate of assessment, when compared to typical trial-and-error methodology [4]. This pipeline has further shown its ability to assess and understand the effects of environmental conditions through the micro- and macroscales [5]. It is clear that further cross-scale exploration, bridging the disciplines of chemistry and geotechnical engineering through the introducing new simple, accessible instrumental tools, offers significant potential to catalyse progression within the field.

When considering previous literature investigating biopolymer soil stabilisation at the microscale, strength improvements have been ascribed to: 1. biopolymer transition from a soft rubbery to glassy state upon drying; 2. the formation of direct hydrogen/electrostatic bonding with fine grained clay particles and 3. the coating (no direct chemical interaction) of coarse grained particles [12, 15, 34, 42]. On the macroscale, frictional and cohesive strength improvements have been attributed to biopolymer-induced inter-particle conglomeration [9, 14, 49, 51]. Biopolymer matrix suction effects have also been hypothesised to contribute to strength characteristics [42]. As of yet, there has been little focus on deciphering the complex biological chemistry which biopolymers present to the biostabilisation field, perhaps due to the field's geotechnical origins. This presents a significant knowledge gap when tailoring biopolymer additives for specific geotechnical applications.

Within this study we therefore aim to explore the effects of key biopolymer characteristics, chemical functionality and molecular weight (M_w_), on the following soil mechanical properties: unconfined compressive strength (UCS), axial strain at peak strength (an indicator of soil ‘ductility’), stiffness and energy absorbance [32]. Energy absorbance (kJ/m^3^) corresponds to the energy required for the creation of deformation within a soil sample and is often used to examine the failure profiles characteristics of cement-stabilised soils and biomaterials such as bone [20, 26].

In order to probe the effect of biopolymer chemical functionality, this study extends previous research. Previous work identified Galactomannan (GM) biopolymers: Locust Bean Gum and Guar Gum to have a ‘high-affinity, high-strength’, specific interactions with Fe_2_O_3_ (Fe) minerals [4]. GM’s are a group of biopolymers derived from plant seed endosperms. Their chemical structure is made up of a β-(1–4)-D-mannan backbone with α-(1–6) D-galactose side chain groups [27]. In solution, galactose (G) side chains exhibit hydrophilic characteristics, whilst mannose (M) backbone groups exhibit hydrophobic characteristics [44, 61]. Depending on the plant source, the galactose side chain substitution, G/M ratio varies from 1:1 to 1:10, making them ideal biopolymer additives to systematically investigate the gradient effects of chemical functionality upon soils mechanical properties [55]. In this study GMs Guar Gum (G:M 1:2), Locust Bean Gum (G:M 1:4) and Cassia Gum (G:M 1:5) were selected for investigation. Throughout this study GM’s are referred to by their G:M ratio.

In order to probe the effect of biopolymer *M*_w_, Carboxy Methyl Cellulose (CMC), a chemically modified cellulose derivative (β-(1–4)-D-glucose backbone), was selected due to the availability of *M*_w_ controlled commercial additives. CMC additives with a *M*_w_ of 90,000 g⋅mol^−1^, 250,000 g⋅mol^−1^ and 700,000 g⋅mol^−1^, were used within this study.

In order to investigate biopolymer chemical functionality and *M*_w_ effects on soils mechanical properties, a soil system has been selected. With at least one major catastrophic tailings dam disaster occurs each year, e.g. the Brumadinho dam failure in 2020, resulted in the release of 11.7 million m^3^ of toxic mine waste mud, causing at least 220 deaths and devastating damage to over 600 km of the Rio Paraopeba River [11], the stabilisation/solidification of MT waste soil material is critical to mitigate these catastrophic failures. Within this study, a simplified MT system based on silica containing 10% iron oxides (by weight) has therefore been selected as a candidate application for biostabilisation. Fe was selected, due to having a universal and consistent abundance (Figure S1), and high relative reactivity in fresh MT conditions [4, 33].

This study aims to demonstrate the critical importance of biopolymer chemistry when applying biopolymer–soil composites within engineering design, a key building block for progression of the field, paving the way towards their use within the next generation of sustainable geotechnical solutions.

## Materials and methods

### Materials and reagents

Guar Gum (G:M 1:2), Locust Bean Gum (G:M 1:4) and Sodium Carboxymethyl Cellulose (CMC), average chain length *M*_w_; 90,000 g⋅mol^−1^, 250,000 g⋅mol^−1^ and 700,000 g⋅mol^−1^ were purchased from Sigma-Aldrich/Merck. Cassia Gum (G:M 1:5) was supplied by Premcem Gums. All biopolymers were used without further purification. Silica Sand (SiO_2_) Fraction E (90–150 μm), a standard reference material for testing cement (BS 1881–131:1998), free from silt, clay & organic matter, was purchased from David Ball sand specialists (Figure S2). Iron oxide (Hematite, Fe_2_O_3_ (Fe)) was acquired from Mineral Waters Ltd and used as supplied.

### Biopolymer additive solution preparation

All biopolymer additive solutions were prepared using the same methodology, determined via preliminary investigations [4]. Solution-biopolymer-preparation, as opposed to soil-biopolymer-preparation, was selected due to GM biopolymers neutral surface charge and hydrophobic characteristics resulting in a high sensitivity towards self-aggregation when ineffectively dispersed, identified in a previous studies preliminary testing [4]. Solution-biopolymer-preparation allows for the better application of dispersion techniques (stirring, temperature, sonication).

**Fig. 1:**
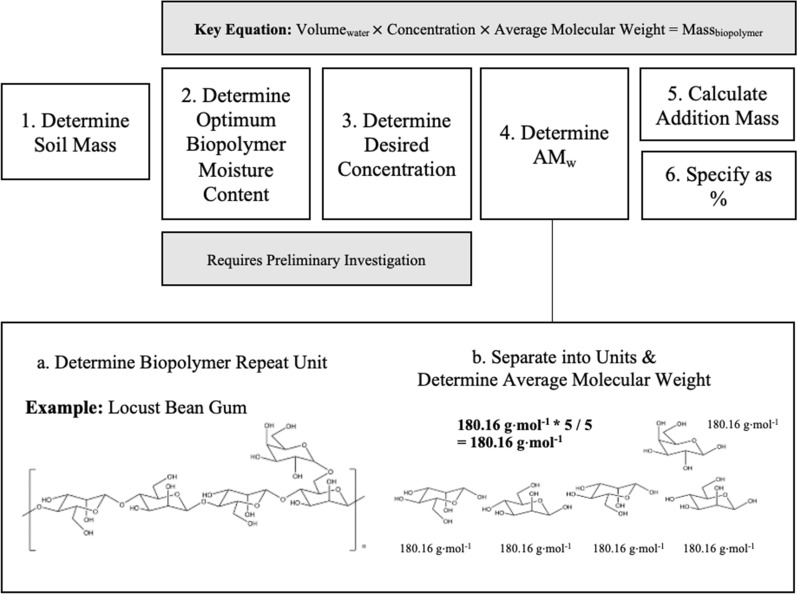
Biopolymer preparation quantities determination steps for geotechnical investigations, including an example of average biopolymer molecular weight (AM_w_) determination using Locust Bean Gum (G:M 1:4)

Powdered biopolymer (GM—1%, CMC—1.44%, Mass_biopolymer_/Mass_soil_) was first added to temperature-controlled (40 °C) ultra-pure water solution (27.5%, Mass_water_/Mass_soil_) whilst simultaneously agitating with a magnetic stirrer (300 rpm). Differing GM and CMC mass quantities were required to achieve equivalent solution molarities (0.2 M, Table 1), utilising average biopolymer molecular weight (AM_w_) (Fig. 1), which is important for comparing bio-mineral binding potential, as outlined within a previous investigation [4]. Solutions were then incubated (10 min, 40 °C) and subsequently sonicated (10 min) using a VWR ultrasonic water bath.

**Table 1:** Summary table of biopolymers preparation quantities for geotechnical testing

	Soil mass (g)	Optimum biopolymer moisture content (ml) (27.5%, Mass _water_/Mass _soil_)	Biopolymer desired concentration (M)	Biopolymer AM_W_(g⋅mol ^−1^)	Biopolymer addition mass (g)	Biopolymer addition percentage (%,Mass _biopolymer_/Mass _soil_)
CMC	160	44	0.2	262.19	2.3	1.44
G:M 1:2	160	44	0.2	180.16	1.6	1
G:M 1:4	160	44	0.2	180.16	1.6	1
G:M 1:5	160	44	0.2	180.16	1.6	1

### Sample preparation

Biopolymer solutions, once prepared, were immediately mixed with 160 g of defined soil matrix and mixed until homogenised. The resulting composite was then divided into 3 equal parts and compacted using a cylindrical drop hammer (2.1103 kg, 246 mm × 37 mm) via 10, 126 mm drops, within a 202 mm × 42 mm hollow cylindrical sample mould. Samples were then extruded and left to cure (7 days, 20 °C). Within this study a SiO_2_ (100%) soil system was investigated, hereby referred to as SiO_2_. A MT exemplar soil system made up of SiO_2_ (90%, by weight) and Fe (10%, by weight), hereby referred to as SiO_2_ + Fe, was also investigated. Each biopolymer–soil combination was performed in triplicate. All sample series SiO_2_ and SiO_2_ + Fe were prepared and cured at the same time to ensure identical curing conditions. The 7-day sample moisture contents and void ratios were in good agreement for all biopolymer-treated samples (7.5–11.5% and 0.47–0.51, respectively (Figure S3)). No significant correlation between moisture retention and UCS was observed within both biopolymer-stabilised SiO_2_ and SiO_2_ + Fe soil systems, indicating no strong influence of suction effects within this study (Figure S4). Previous studies have also shown a lack of correlation between suction and biopolymer-stabilised soils compressive characteristics [42].

### Geotechnical verification (GV)

A digital Tri-test ELE was used to perform UCS tests following the ASTM D2166 standard method [29]. A rate of displacement of 1.5 mm min^−1^ was utilised throughout all testing, and load and displacement data were collected. Sample bedding errors were removed pre-data analysis. The UCS at failure of each sample was determined as the peak applied axial load, per cross-sectional area. Axial strain at peak strength was determined as the sample vertical displacement at failure as a proportion of the original sample height. Secant stiffness at failure was determined via load/displacement at failure. Sample height, weight and mass were taken over the 7 days to determine moisture content retention and final soil void ratio.

### Mineral binding characterisation (MBC)

MBC was performed in order to decode the microscale interactions driving bio-mineral interactions [4]. Fe particles were the focus of this study due to their higher relative surface reactivity. 0.01 M solutions of each biopolymer were prepared in ultra-pure water (20 ml) via the methodology previously outlined (2.2.). Fe (64 mg, 0.02 M) particles were then added and dispersed via sonication (10 min, VWR ultrasonic water bath). The solution pH was then adjusted to pH 7 using NH_4_OH (0.5 M)/HCl (0.5 M) where necessary. The solutions were then rotated for 30 min using a Lab net Mini Labroller™. Biopolymer-coated particles were separated using centrifugation (4000 rpm, 10 min) and washed using ultra-pure water to remove excess non-bound biopolymers (4 repeats). Particles were then left to dry at room temperature, ready for analysis.

Particle organic coating masses were determined using a Perkin Elmer Pyris 1 Thermal Gravimetric Analyzer (TGA). Dry Bio-Fe_2_O_3_ particles were exposed to a temperature range of 20–800 $^\circ{\rm C}$ under a 2/3 N_2_, 1/3 O_2_ atmosphere. Biopolymer mass loss (%) was determined between 200 and 400 $^\circ{\rm C} .$

Zeta potentials were determined using a Brookhaven BI-900AT. Bio-Fe_2_O_3_ particles were ground using a pestle and mortar and dispersed (0.01 mg/ml) via sonication (10 min, VWR ultrasonic water bath) in a KNO_3_ (10 mM) solution. The solution pH was adjusted using NaOH (0.5 M)/ HCl (0.5 M) to pH 7. Samples were scanned 5 times at 25 $^\circ{\rm C}$ and data analysed using Malvern ZetaPlus software.

Surface functional groups were determined using a Perkin Elmer Frontier Fourier Transform Infrared (FTIR) and Golden Gate Diamond Attenuated Total Reflection (ATR) spectrometer. Data collection and analysis was performed using Spectrum™ 10. Scans were made between 4000 and 400 cm^−1^. Baseline correction was performed on all spectra.

## Results

### Biopolymer ***M***_w_ effects on soil mechanical properties

**Fig. 2:**
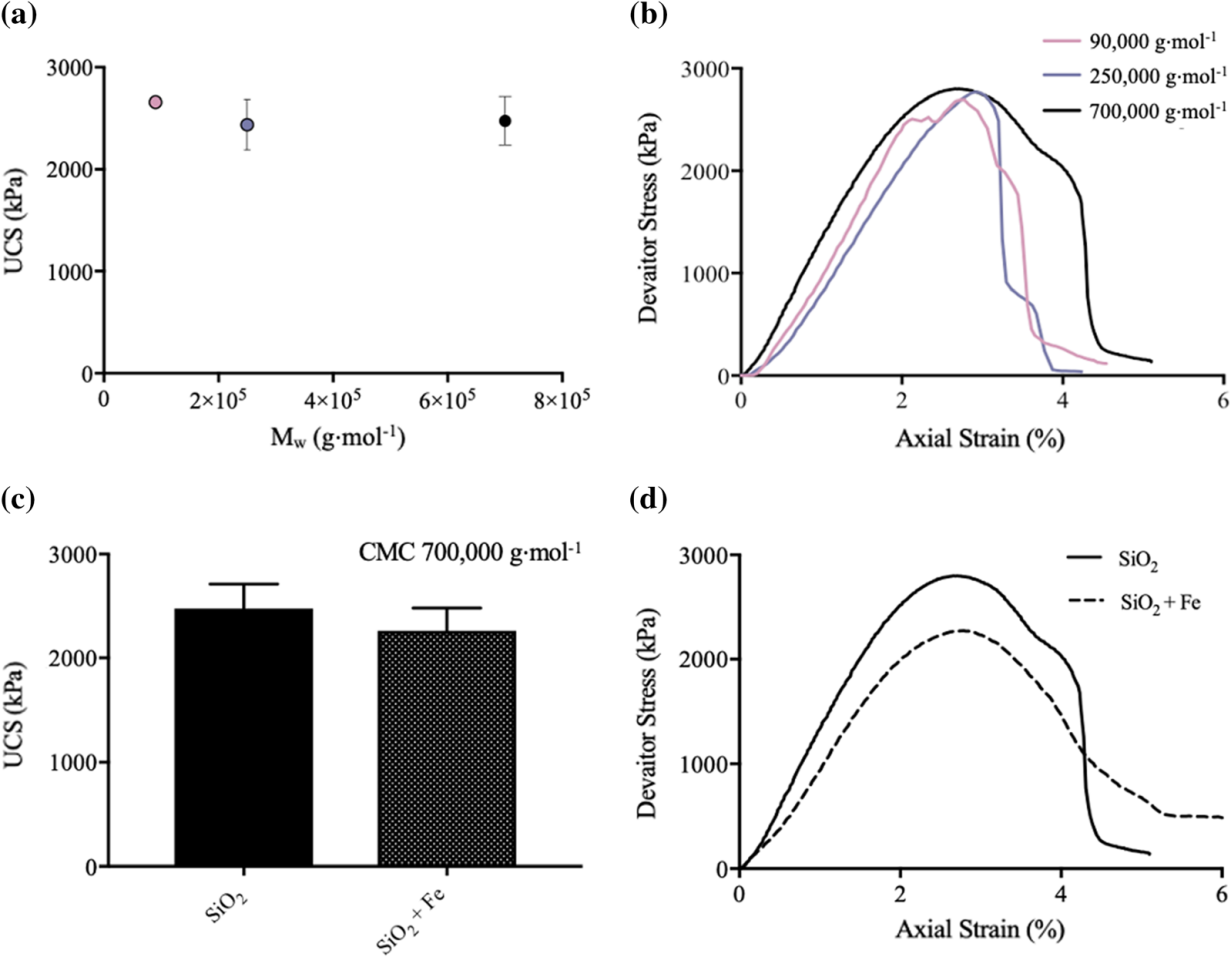
The effects of CMC upon soil strength characteristics, after 7 days of curing at 20 $^\circ{\rm C}$. **a** UCS (kPa) at failure of SiO_2_ upon increasing biopolymer *M*_w_ (90,000–700,000 g⋅mol^−1^). **b** Stress–strain curves of CMC (90,000, 250,000, 700,000 g⋅mol^−1^)-stabilised SiO_2_. **c** UCS (kPa) at failure of SiO_2_ and SiO_2_ + Fe soil systems upon the addition of CMC (700,000 g⋅mol^−1^). **d** Stress–strain curves of CMC (700,000 g⋅mol^−1^)-stabilised SiO_2_ and SiO_2_ + Fe

UCS tests were performed in triplicate on SiO_2_ soils stabilised with CMC of varying average chain length, to determine the effect of *M*_w_, a key biopolymer characteristic, upon soil strength properties. Upon an increase in *M*_w,_ a non-significant change in UCS (Fig. 2a), axial strain at peak strength (Figure S5A) and stiffness (Figure S5B) was observed. When examining stress–strain curves (Fig. 2b) an increase in total energy absorbed, with increasing *M*_w_, was exhibited, with improvements predominantly arising from post-failure energy absorption.

CMC (700,000 g⋅mol^−1^) stabilisation was investigated within a SiO_2_ + Fe soil system. With addition of Fe (10%), a non-significant change in UCS at failure was observed (Fig. 2c). Furthermore, analysis of stress–strain curves shows little change in overall strength characteristics (Fig. 2d). This is postulated to be due to the presence of deprotonated carboxyl groups at pH 7, resulting in a lack of specific interactions with the Fe mineral surface, as found in previous investigations [4]. Due to this poor affinity, CMC (700,000 g⋅mol^−1^) has been used as a positive control within chemical functionality investigations.

### Biopolymer chemical functionality effects on soil strength

**Fig. 3:**
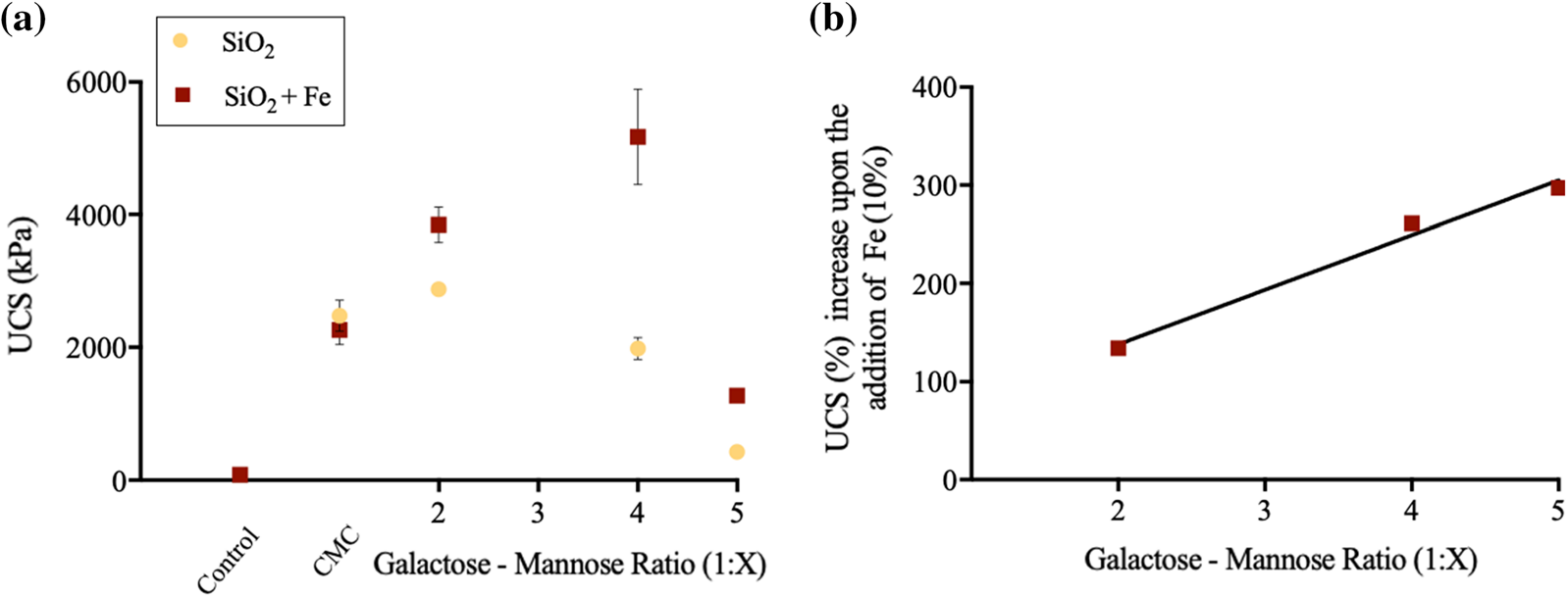
Biopolymer chemical functionality effects upon soil strength properties. **a** UCS (kPa) at failure of GM (1%, Mass_biopolymer_/Mass_soil_), (G:M, 1:2–1:5), stabilised SiO_2_ and SiO_2_ + Fe soil systems after 7-day curing at 20 $^\circ{\rm C}$. CMC (1.44%, Mass_biopolymer_/Mass_soil_) has been used as positive control following the identification of its lack of Fe affinity (Fig. 2). SiO_2_ control exhibited a negligible UCS due to the lack of cohesional strength. **b** UCS (%, (Average UCS_SiO2+Fe_ /Average UCS_SiO2_) × 100) increase upon the addition of Fe (10%), when increasing mannose content, G:M 1:2–1:5

When compared to mineral control samples, the addition of GM biopolymers (1%, Mass_biopolymer_/Mass_soil_) resulted in dramatic improvement in UCS for all samples (Fig. 3a).

Upon the addition of GM (1:2) to SiO_2_ samples, a UCS of 2878 kPa is observed. With increased mannose content, G:M 1:2 to G:M 1:5, a negative correlation is seen, with UCS reducing by 85% (2878–429 kPa).

When examining GM-stabilised SiO_2_ + Fe, relative to SiO_2_ soil systems, strength gains in all GM-stabilised samples are seen, with an improvement of 970 kPa, 3190 kPa and 848 kPa for 1:2, 1:4 and 1:5 G:M, respectively. Peak strength was achieved for G:M 1:4 samples (5171 kPa).

Although UCS improvements peaked at GM 1:4 ratio, for the SiO_2_ + Fe soil system, one should also consider the relative increase (Average UCS_SiO2+Fe_ /Average UCS_SiO2_ × 100) between the SiO_2_ and the SiO_2_ + Fe as this gives a better indication of the role of iron binding, removing the effects of GM—SiO_2_ binding. A direct and fairly linear correlation between G:M ratio and % strength gain is exhibited, with G:M 1:5 exhibiting a 296% increase (Fig. 3b).

### Biopolymer chemical functionality effects on further soil mechanical properties

Relative to control samples, all biopolymer-stabilised samples showed significant improvements in all examined mechanical properties.

**Fig. 4:**
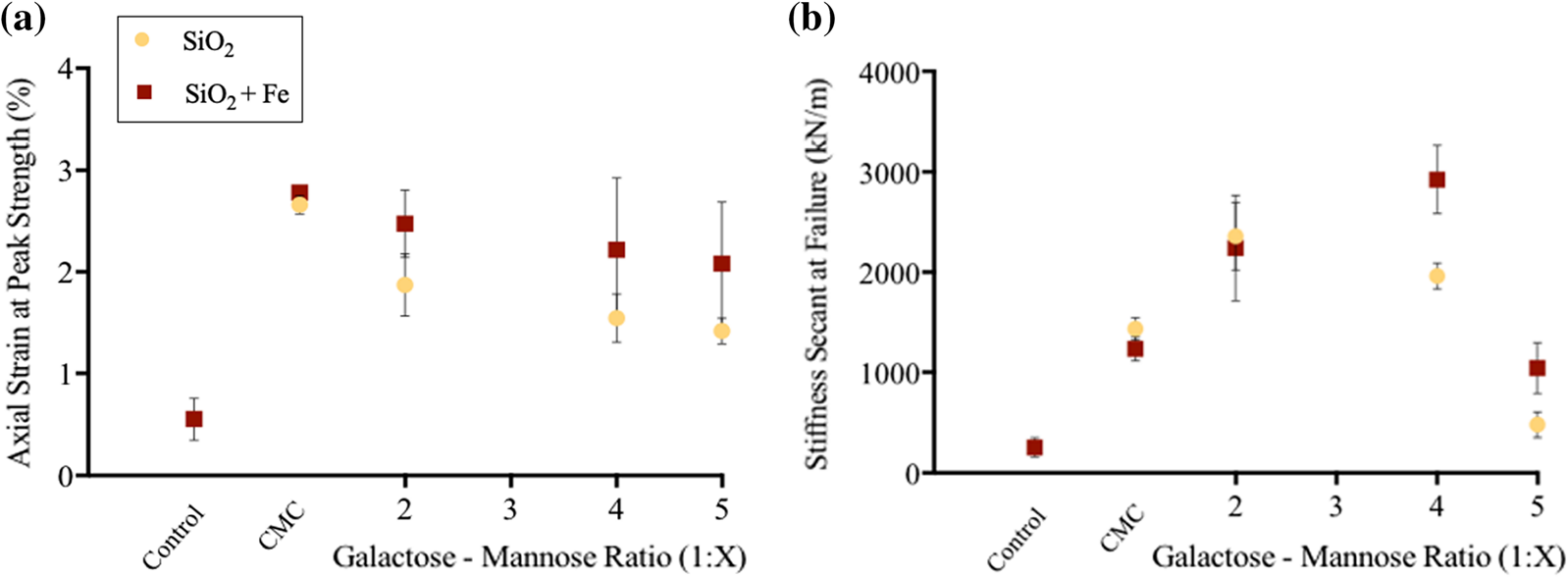
Biopolymer (GM, CMC) functionality effects upon further soil (SiO_2_, SiO_2_ + Fe) mechanical properties. CMC has been used as positive control following the identification of its lack of Fe affinity (Fig. 2). **a** Axial strain (%) at peak strength on increasing mannose content, G:M 1:2–1:5. **b** Stiffness secant at failure (kN/m) on increasing mannose content, G:M 1:2–1:5

**Fig. 5:**
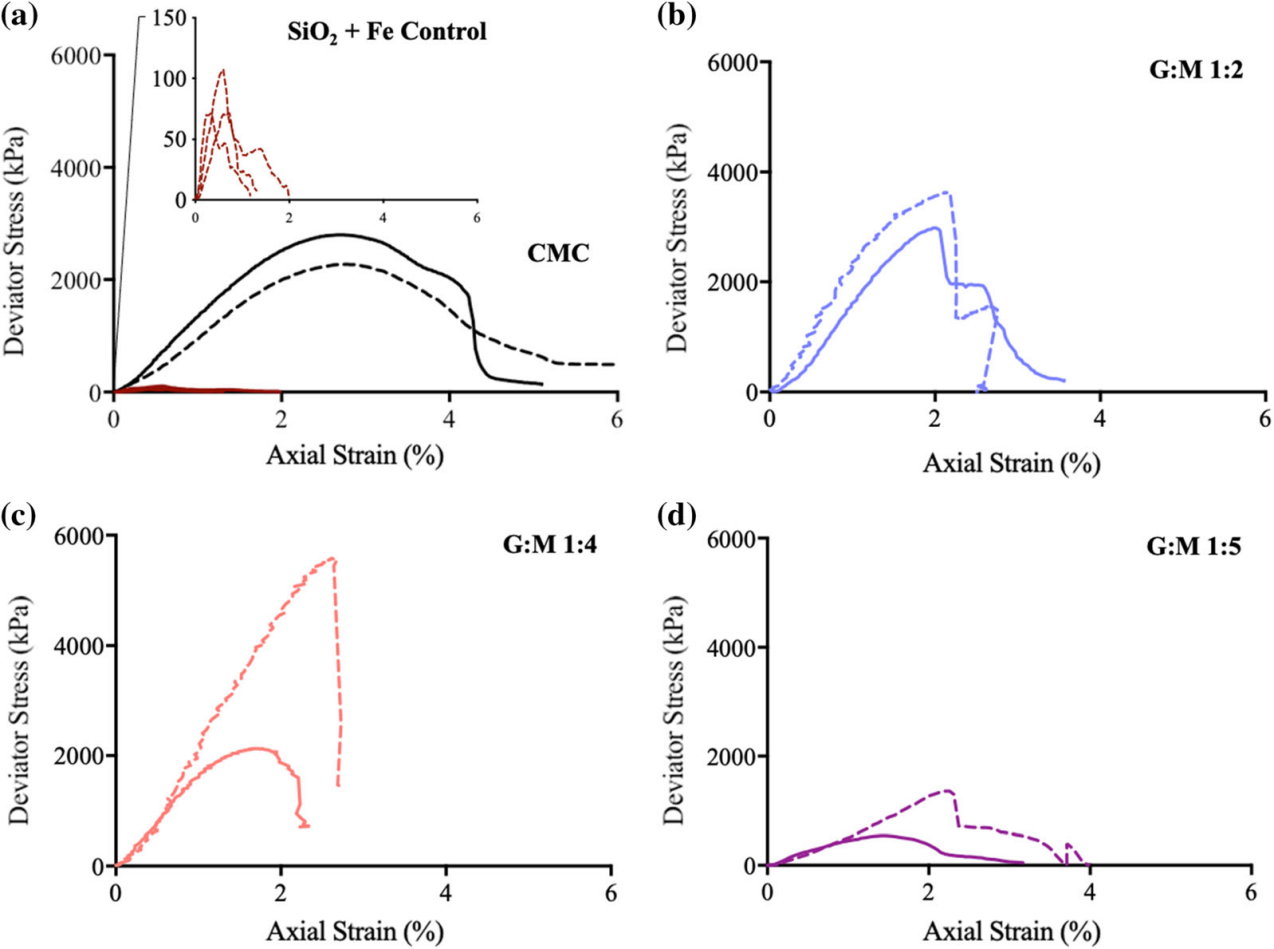
Stress–strain profiles of biopolymer-stabilised SiO_2_ (bold line) and SiO_2_ + Fe (dotted line) samples after 7-day curing at 20 $^\circ{\rm C}$. **a** SiO_2_ + Fe control and CMC-stabilised positive controls. SiO_2_ exhibited negligible UCS due to the lack of cohesional strength; therefore, no stress–strain profile was recorded. **b** G:M 1:2. **c** G:M 1:4 **d** G:M 1:5

Notably, GM-stabilised samples show a negative linear relationship between G:M (1:2–5) ratio and axial strain at peak strength for SiO_2_ and SiO_2_ + Fe (Fig. 4a). As found in previous studies [42], stiffness value trends (Fig. 4b) show a slight deviation from UCS values. When further considering stress–strain curves (Fig. 5), notably higher pre-failure–low post-failure energy absorbance was observed within G:M 1:2 and 1:4 stabilised SiO_2_ + Fe, when compared to other GM-stabilised samples.

### Mineral binding characterisation (MBC) of GM-Fe

MBC (TGA, Zeta Potential and ATR-FTIR) was carried out on GM-coated Fe particles to determine microscale bio-mineral interactions driving soil mechanical property improvements.

**Fig. 6:**
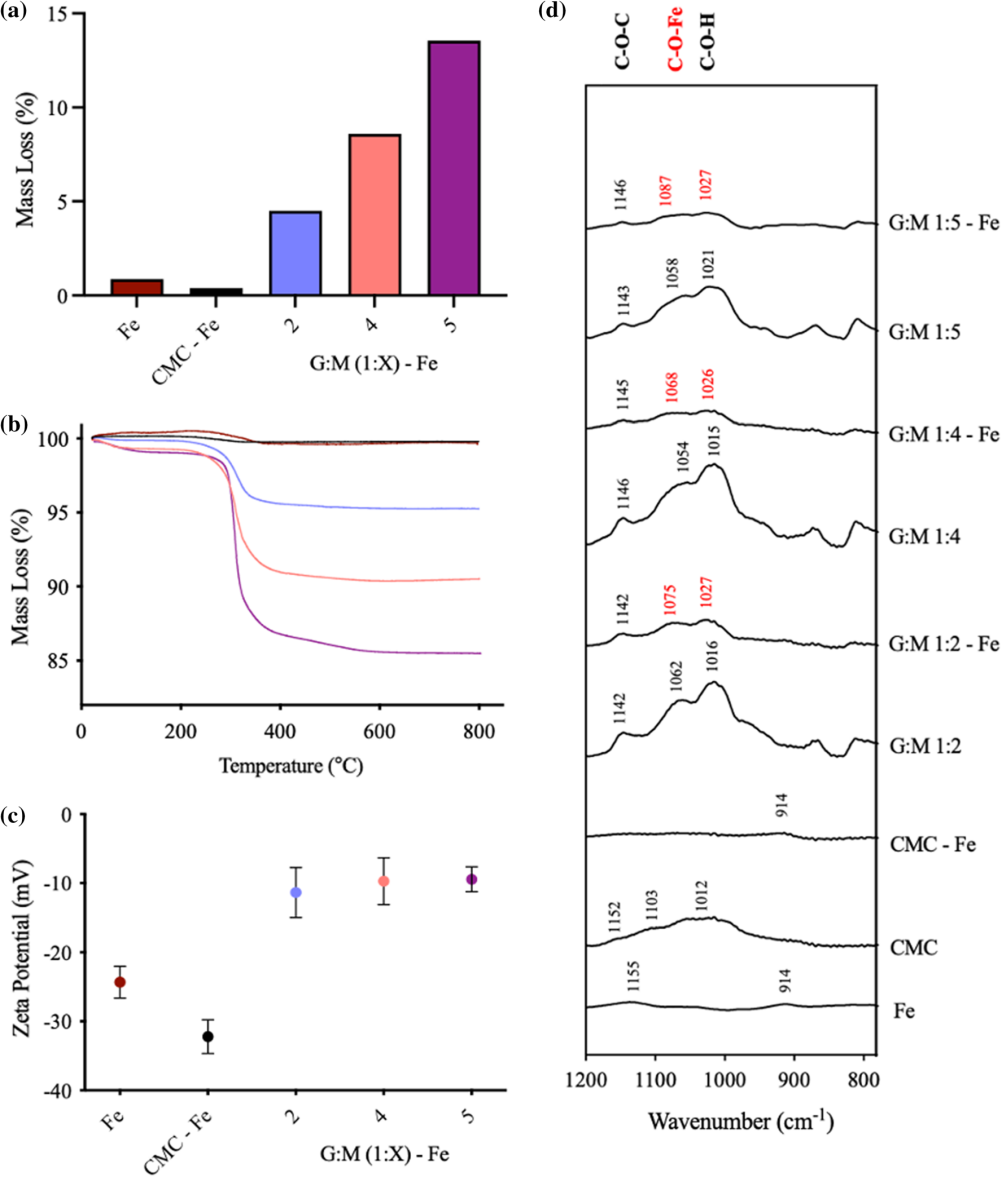
Mineral binding characterisation (MBC) **a** MBC—TGA of Bio-Fe particles showing the mass loss (%) upon a temperature gradient (200–400 $^\circ{\rm C}$). **b** TGA of Bio-Fe showing mass loss (%) over full temperature gradient (200–800 $^\circ{\rm C}$). **c** Zeta potential (mV) of dispersed (0.01 g/ml) Bio-Fe particles within a KNO_3_ (10 mM) solution at pH 7. **d** ATR-FTIR showing the absorbance of Bio-Fe as a function of wavenumber (1200–780 cm^−1^), baseline corrections have been performed

TGA (Fig. 6a) indicated a direct correlation between G:M ratio and Fe binding affinity, with GM 1:5 exhibiting the highest biopolymer mass loss (13.5%, Fig. 6a, b). The shift of biopolymer C–O–H groups within the ATR-FTIR spectra (Fig. 6d) indicates the formation of covalent C–O–Fe bonds. Little presence of biopolymer additive was observed within CMC-Fe spectra. An increase in surface charge to within the threshold of aggregation (− 15mv) of all GM-Fe particles (Fig. 6c) indicates an electrostatic component to the binding mechanism. These results indicate that high affinity GM-Fe is driven by a combination of direct covalent C–O–Fe and electrostatic surface interactions, determined by their G:M ratio.

## Discussion

### Soil mineralogy effects on soil strength properties

Within this study two soil systems have been investigated; SiO_2_ and SiO_2_ + Fe. Within neutral (pH 7) aqueous solutions, SiO_2_ has a high negative surface charge (− 75 mv) resulting in strong electrostatic repulsive forces [52]. These repulsive forces are likely associated with a lack of cementitious interactions, and therefore cohesive strength, between SiO_2_ particles, resulting in negligible UCS within mineral control samples (Fig. 3). The minor improvements in UCS observed within the SiO_2_ + Fe are likely due to smaller negative surface charge (− 26 mv) associated with Fe particles [4].

### Biopolymer chemical functionality effects on SiO_2_ strength

Within SiO_2_ soil samples, peak UCS values were exhibited within G:M 1:2 ratio additives (2878 kPa) (Fig. 3). Upon increasing mannose content, G:M 1:2–5, a 85% reduction in strength is seen, with G:M 1:5 exhibiting the lowest UCS (429 kPa). It is proposed that the microscale G:M chemical characteristic plays an important role in the dramatic reduction in UCS.

**Fig. 7:**
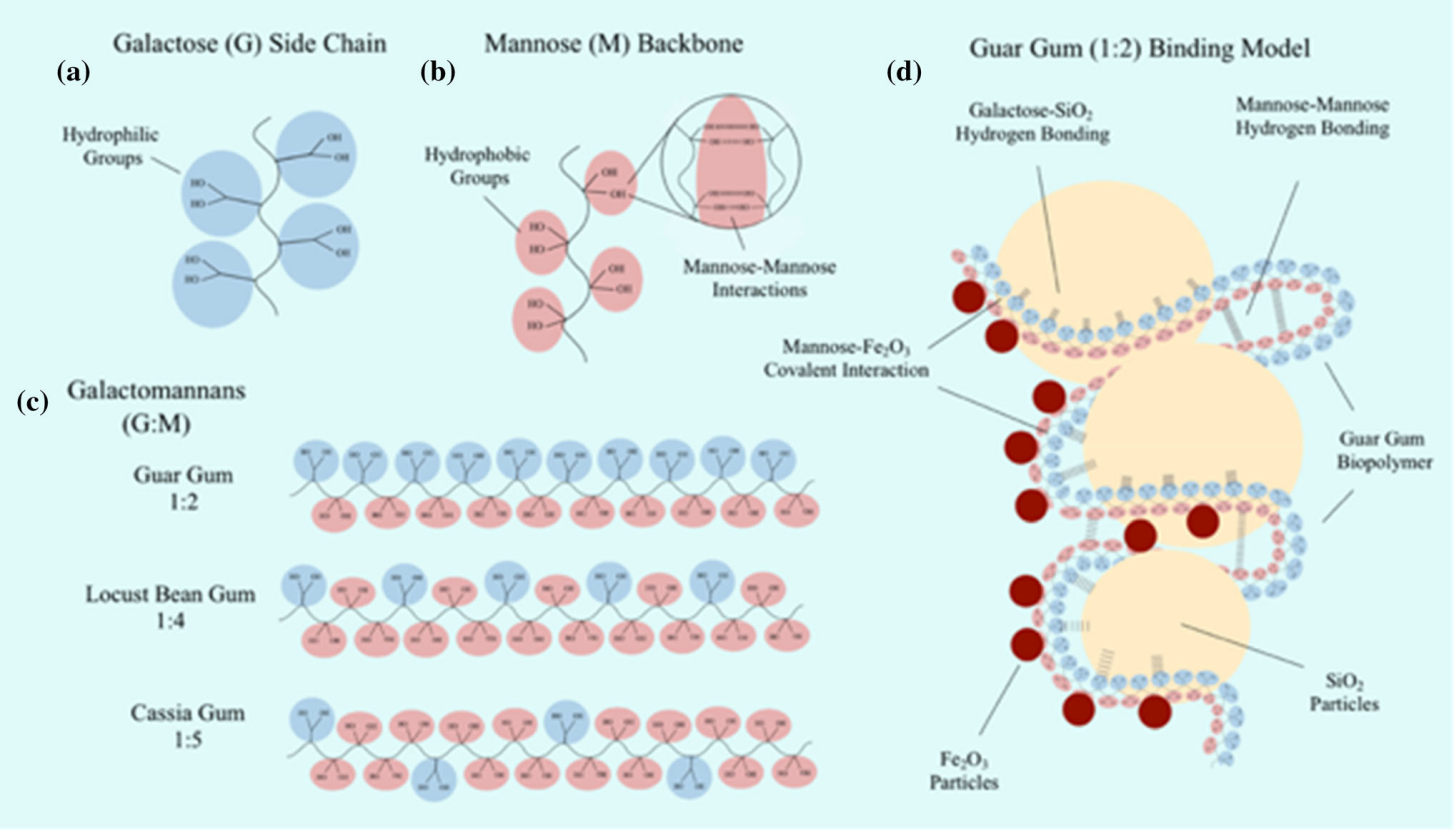
Bio-mineral GM binding model schematic. **a** Simplified representation of hydrophilic galactose side chain groups. **b** Simplified representation of hydrophobic mannose backbone groups showing high affinity intermolecular bio-bio interactions. **c** Galactomannan simplified representation showing the ratio of hydrophilic galactose group and hydrophobic mannose groups in G:M 1:2, G:M 1:4 and G:M 1:5. **d** G:M 1:2 simplified binding model, displaying galactose-SiO_2_ hydrogen bond-mediated interactions, mannose-Fe covalent interactions and mannose–mannose intermolecular interactions (components not to scale)

Galactose groups, due to their hydrophilicity (Fig. 7a), have the ability to form non-specific water-assisted hydrogen bond interactions with the SiO_2_ surface [38, 39]. In contrast, due to their hydrophobic characteristic (Fig. 7b), mannose groups are unable to form water-assisted, hydrogen bond interactions with the SiO_2_ surface. The 85% reduction in strength is therefore attributed to the increased proportion of mannose groups in G:M 1:5 (Fig. 7c), with the inability to form bio-mineral interactions, resulting in a lack of effective load distribution.

### Biopolymer chemical functionality effects on SiO_2_ + Fe strength

Within GM-stabilised SiO_2_ + Fe soil systems, when considering UCS improvements upon the addition of Fe (10%) (in isolation of the negative effects observed with SiO_2_), a positive correlation between G:M ratio and relative UCS improvement is observed, peaking at G:M 1:5 (297%) (Fig. 3b). An identical trend is observed within microscale MBC experiments, with MBC:TGA showing a direct correlation between G:M ratio and Fe binding potential (Fig. 6a, b). When probing the chemical surface functional groups, MBC: ATR-FTIR (Fig. 6d) a loss of intensity and peak shift associated with C–O–H groups is seen, indicating their conversion to C-O-Fe bonds, as found in previous studies [4, 31, 41]. MBC: Zeta potential further highlights the presence of electrostatic contribution to microscale driving forces, as upon GM addition, surface charge shifts to within the threshold of aggregation (Fig. 6c). This microscale information indicates that the strength improvements observed, are due to increased proportion of mannose groups, with the ability to form ‘high-affinity, high-strength’ bio-mineral (mannose—Fe) interactions.

When considering both SiO_2_ and Fe contributions, UCS peak is found with G:M 1:4 stabilised SiO_2_ + Fe (Fig. 3a). This is attributed to the ability of LB to form both a high proportion of hydrogen bond-mediated galactose-SiO_2_ interactions and specific covalent mannose-Fe interactions, resulting in synergistic interactions and a higher overall UCS. A qualitative bio-mineral binding model has been constructed representing the microscale interactions associated with the G:M-stabilised SiO_2_ + Fe soil system (Fig. 7d).

**Fig. 8:**
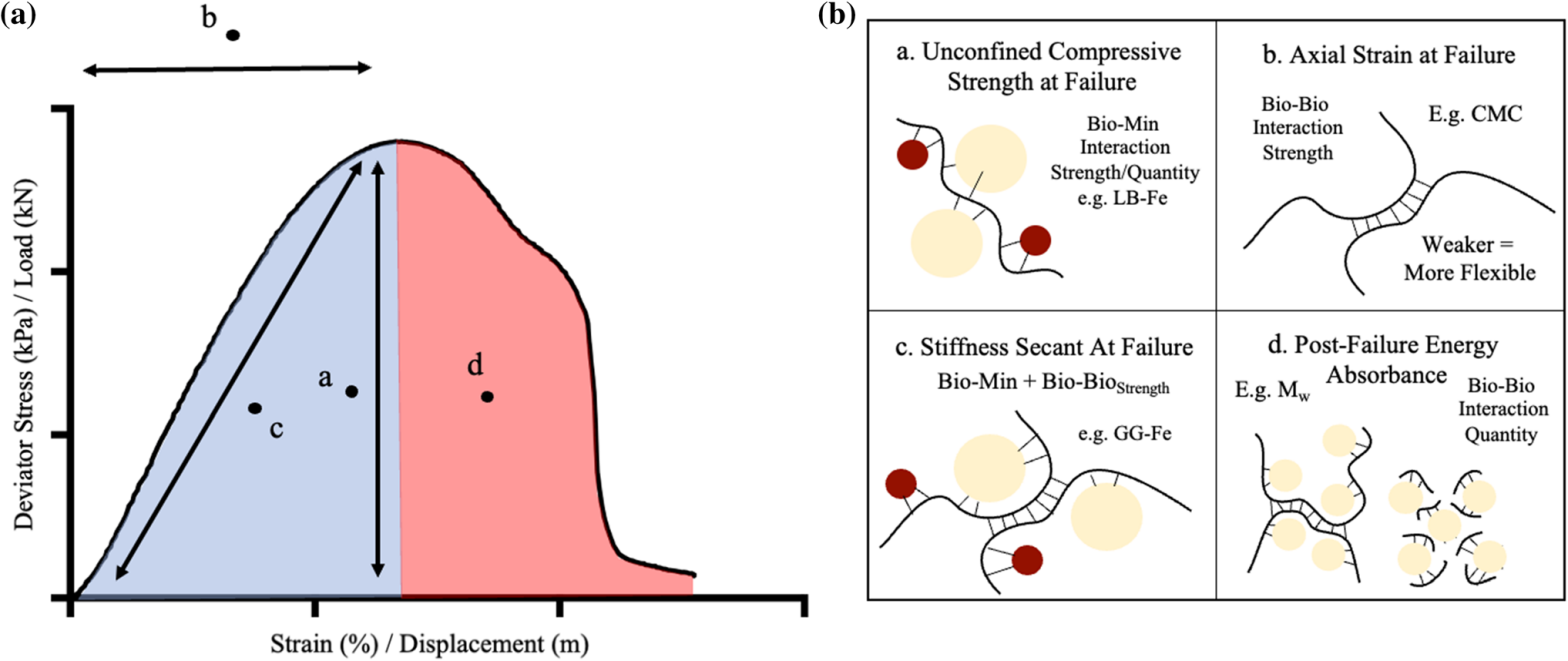
Biopolymer soil stabilisation strength characteristics summary schematic. **a** An example deviator stress (kPa)/load (kN)–axial strain (%)/displacement (m) graph, highlighting important strength characteristics associated with biopolymer-stabilised soils. **b** Summary table showing the proposed important biopolymer characteristics associated with each strength property represented on a stress–strain profile (bio-mineral representation components not to scale); a. the importance of bio-mineral interaction strength and quantity on the unconfined compressive strength (kPa) at failure, b. The importance of bio-bio interaction strength on the axial strain (%), c. The importance of both bio-mineral and bio-bio interaction strength on stiffness (kN/m), d. The importance of bio-bio quantity on post-failure energy absorbance (kJ/m^3^)

The critical importance of bio-mineral interactions upon the resulting macroscopic compressive strength properties has been determined (Fig. 8), with mannose group’s inability to bind to the SiO_2_, but propensity to form ‘high-affinity, high-strength’ GM-Fe interactions, resulting in significant soil strength implications.

### Biopolymer additive characteristics effects on further soil mechanical properties

#### Axial strain at peak strength

Although strength is a critical parameter when it comes to soils use within an engineering application, the ability to modify additional mechanical properties is critical for biopolymers use as widespread soil stabilisation additives.

When considering the axial strain at peak strength properties of biopolymer-stabilised soils a number of trends have been observed. Within both GM-stabilised SiO_2_ and SiO_2_ + Fe systems, upon increasing mannose content, a negative correlation is observed with axial strain at peak strength (Fig. 4a). Within previously investigated bio-mineral material systems, deformation ability has been attributed to the soft ductile nature of the biopolymer component, determined by the *strength* of bio-bio intermolecular interactions [35, 37, 47]. As GM biopolymer rigidity has previously been attributed to strong intermolecular hydrogen bonding between mannose groups [19], it is therefore postulated that the lower axial strain at peak strength values observed for G:M 1:5 is due to an increased proportion of high *strength* intermolecular bio-bio (mannose-mannose) interactions.

On increasing *M*_w_ CMC-stabilised samples exhibit a non-significant change in axial strain at peak strength (Figure S5A). However, CMC-stabilised samples exhibit the highest axial strain at peak strength values recorded in this study (2.4–3%). It is postulated that this is due to CMC’s extended biopolymer structure at pH 7, resulting from repulsions by negatively charged carboxyl groups. This results in relatively *weaker* bio-bio interactions and therefore an increased ability of the soil to absorb deformation [21]. It is clear that, as found within previous natural bio-mineral composites, bio-bio intermolecular interaction *strength* plays an important role in the ductility of biopolymer-stabilised soils (Fig. 8).

#### Stiffness secant at failure

When considering UCS and axial strain at peak strength, little correlation is observed, indicating an independence between bio-mineral (UCS) and bio-bio (axial strain) property contributions. This would account for unexplained differences between UCS and stiffness (resistance of a soil to deform under load), highlighted within this study (Figs. 3a, 4b) and previous investigations [42]. Therefore it is important to consider both bio-mineral and bio-bio interactions when considering a biopolymer-stabilised soils stiffness (Fig. 8).

#### Energy absorbance

On reviewing GM-stabilised soil stress–strain curves (Fig. 5), a notably high proportion of pre failure energy absorbance is observed within G:M 1:2 and G:M 1:4 stabilised SiO_2_ + Fe soil samples. It is postulated that this is derived from the predominance of bio-mineral interactions (galactose-SiO_2_ and mannose-Fe) within these soil systems.

Despite little change in UCS, axial strain at peak strength and stiffness, when increasing CMC M_w_ from 90,000 to 700,000 g⋅mol^−1^, an increased total energy absorption is observed within stress–strain curves (Fig. 2b). Increases are further seen to be predominantly derived from post-failure absorption characteristics. It is hypothesised that the increased post-failure energy absorption is derived from a strain softening mechanism, due to the increased *quantity* of bio–bio intermolecular interactions associated with long-chained biopolymers. Strain softening mechanisms such as sacrificial polymer bonds and a chain lengthening, have been seen in bio-mineral systems within nature [2, 18]. For example, a study by Murcia et al. [43] found a 100% increase in total energy absorption associated with a bio-mineral composite fish scale material, associated with increased *quantity* of interpeptide bio-bio hydrogen bonding, supporting this hypothesis. Therefore, this study has highlighted the importance of bio-bio interaction *quantity* when investigating biostabilisation of soils (Fig. 8).

### Biopolymer–soil mix design principles for future geotechnical investigations

Through deciphering the effects of key biopolymer characteristics, chemical functionality and molecular weight, on geotechnical properties, design principles can be extrapolated. This study has shown that when utilising charged/hydrophilic minerals, such as SiO_2_ (at pH 7), there is a greater capacity to form non-specific electrostatic bio-mineral interactions, whilst more neutral/hydrophobic minerals, such as Fe (at pH 7), have a greater capacity to form specific covalent bio-mineral interactions. The same principle applies to biopolymer functional groups, with charged/hydrophilic biopolymer groups, such as galactose, containing a greater capacity to form electrostatic bio-mineral interactions and more neutral/hydrophobic groups, such as mannose, a greater capacity to form covalent interactions [23].

This study has also highlighted that bio–bio interactions are preferable when contrastingly charged biopolymer and mineral constituents are exposed to one another (e.g. galactose-SiO_2_), resulting in a reduction in UCS improvements. Therefore when maximal soil UCS is desired, complimentary biopolymer and mineral surface energetics should be considered, whilst, when tailoring deformation characteristic, contrasting biopolymer and mineral surface energetics should be examined. This study has further highlighted that it is also important to consider both backbone and side chain functionality when considering the potential bio-mineral interactions with a soil system. The effect of functional group availability, due to biopolymer structural effects (such as gelation), has previously been shown as an important chemical characteristic to also consider [4].

Although understanding of ‘bottom-up’ microscale fundamentals as addressed in this paper can provide biopolymer interaction indicators, the alternative or additional use of simple, high-through-put methodologies, such as Membrane Enabled Bio-mineral Affinity Screen (MEBAS), can also allow for the identification of high affinity bio-mineral composites, without the need for comprehensive microscale understanding [4].

**Fig. 9:**
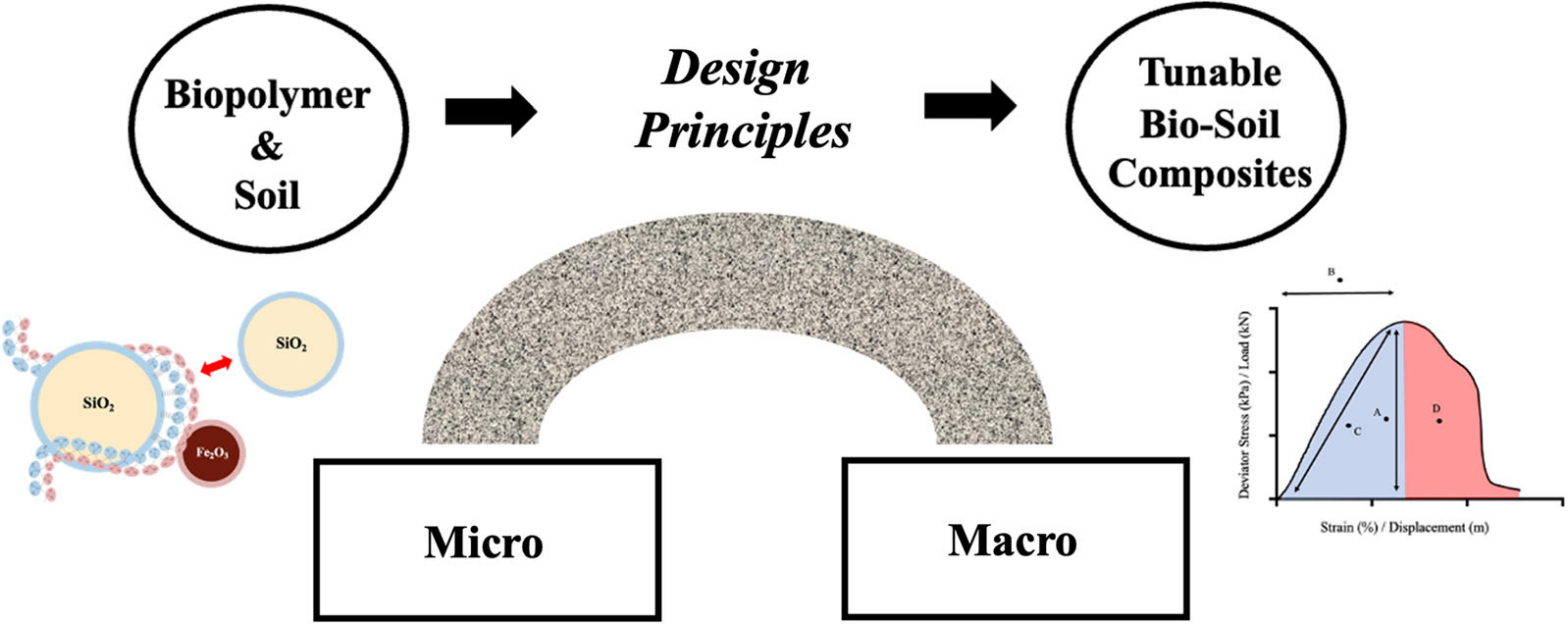
Schematic highlighting the importance of design principles for the bridging of micro- and macroscales, allowing the production of tuneable biosoil composites, catalysing progression within the field of biopolymer soil stabilisation

A multitude of research avenues has arisen from this study. The use of a biopolymer-orientated approach to investigate the vast catalogue of biopolymers in diverse, real-world, problematic soil systems (e.g. soft clay), will allow the building of a database of synergistic biopolymer–soil composites, strengthening the foundations of the field. The further introduction of additional micro-scale experimental and non-experimental tools, widening the bridge between the ‘micro and macro scales’, will improve engineers predictive capabilities. Finally, the translation of the design principles outlined within this study to investigate further properties (e.g. durability), will expand the application potential of biopolymer–soil composite use within engineering design (Fig. 9).

## Conclusions

The effects of Galactomannan (GM) biopolymer chemistry (chemical functionality, molecular weight), on the mechanical properties of a SiO_2_, Fe_2_O_3_ soil have been investigated. Theoretical considerations confirmed by mineral binding characterisation (MBC) demonstrated that the GM mannose group has an inability to bind to SiO_2_, but propensity to form ‘high-affinity, high-strength’ GM-Fe interactions, whereas in contrast the GM galactose group has a higher affinity to SiO_2_. Macroscopic compressive strength tests demonstrated strength variations by up to a factor of 12 across the sample mixes studied and in line with the variation expected due to the differences in the galactose/mannose ratios. The limited impact of molecular weight upon soil strength properties has also been shown in CMC-stabilised soils. Notably, the previously unidentified importance of biopolymer–biopolymer (bio–bio) intermolecular interactions when considering soil’s ability to deform (axial strain at peak strength), resist deformation (stiffness) and absorb energy (toughness) has been identified, introducing an important further considerations to the field.

Through this study’s findings a number of geotechnical mix design principles have been determined. When maximal soil UCS is desired, complimentary biopolymer and mineral surface energetics should be considered, whereas when tailoring deformation characteristics, contrasting biopolymer and mineral surface energetics should be examined. The further delineation of non-specific electrostatic interactions between charged/hydrophilic constituents and specific covalent interaction between neutral/hydrophobic constituents, will provide key foundational understanding for the investigation and application of biopolymer–soil composites.

The potential of a simple, low-cost, accessible, chemistry-based experimental tools has also been illustrated, providing a basis for future biopolymer-orientated investigations. Whilst there remain areas of biopolymer–soil interaction to be investigated, these findings contribute to the expanding knowledge base needed to enable robust biopolymer use within the next generation of sustainable geotechnical solutions.

## Supplementary Information

Below is the link to the electronic supplementary material.

Typical Mine Tailing (MT) composition (Figure S1); table showing SiO2 properties (Figure S2); moisture retention and void ratios of biopolymer-stabilised soil systems after 7-day curing at 20℃ (Figure S3); relationship between UCS and moisture retention of biopolymer-stabilised soil systems after 7-day curing at 20℃ (Figure S4); further mechanical properties of CMC-stabilised SiO2 (100%) samples upon increasing molecular weight (Figure S5) (PDF). (PDF 352 kb)

## Data Availability

The datasets generated during and/or analysed during the current study are available from the corresponding author on reasonable request.

## Abbreviations

AM_w_: Average biopolymer molecular weight
ATR: Attenuated total reflectance
CMC: Sodium carboxy methyl cellulose
Fe: Fe_2_O_3_
FTIR: Fourier transform infrared spectroscopy
G: Galactose
GM: Galactomannan
G:M 1:2: Guar gum
G:M 1:4: Locust bean gum
G:M 1:5: Cassia gum
GV: Geotechnical verification
M: Mannose
MEBAS: Membrane enabled bio-mineral affinity screen
MBC: Mineral binding characterisation
MT: Mine tailing
Mw: Molecular weight
SiO_2_: SiO_2_ (100%, by weight)
SiO_2_ + Fe: SiO_2_ (90%, by weight) + Fe_2_O_3_ (10%, by weight)
TGA: Thermal gravimetric analysis
UCS: Unconfined compressive strength
