# Supplementary material for: Sustainable biopolymer soil stabilisation: the effect of microscale chemical characteristics on macroscale mechanical properties
Source: Acta Geotech. 2022 Dec 11;18(6):3213–27. doi: 10.1007/s11440-022-01732-0 (PMC10264278; doi:10.1007/s11440-022-01732-0)
Supplement: Supplementary file 1 — Typical Mine Tailing (MT) composition (Figure S1); table showing SiO2 properties (Figure S2); moisture retention and void ratios of biopolymer-stabilised soil systems after 7-day curing at 20℃ (Figure S3); relationship between UCS and moisture retention of biopolymer-stabilised soil systems after 7-day curing at 20℃ (Figure S4); further mechanical properties of CMC-stabilised SiO2 (100%) samples upon increasing molecular weight (Figure S5) (PDF). (PDF 352 kb) [file 11440_2022_1732_MOESM1_ESM.pdf]

Supplementary Information

Sustainable Biopolymer Soil Stabilisation – The Effect of Microscale Chemical Characteristics on Macroscale Mechanical Properties

Samuel J. Armistead,[1,2] Colin C. Smith,[2*]
Sarah S. Staniland[1*]

[1]Department of Chemistry, The University of Sheffield, Dainton building, Brook Hill, Sheffield S3 7HF, UK

[2]Department of Civil and Structural Engineering, The University of Sheffield, Sir Frederick Mappin Building, Mappin Street, Sheffield, S1 3JD, UK

* Corresponding authors: Correspondence should be sent to c.c.smith@sheffield.ac.uk and s.s.staniland@sheffield.ac.uk

S1. Typical Mine Tailing (MT) Ore, Mineral Composition & pH.

Mine Tailing Site Location	Ore	Fe_2O_3	Al_2O_3	SiO_2	CaO	pH	Reference
China (A)	Fe	14.37	0.8	82.26	0.57	-	[1]
China (B)	Fe	12.61	1.65	75.46	1.70	-	[2]
South Africa (A)	Au	11.7	14.17	58.12	7.5	8.0	[3]
South Africa (B)	Cu	8.07	12.06	65.5	5.91	8.5	[4]
Tanzania	Au	10.40	9.61	50.74	5.34	7.2-7.5	[5]

S2. Table showing Silica Sand (SiO_2) properties conforming to BS 1881-131-1998 for testing cement.

	Fraction E
**Size**	150 μm – 90 μm
**Min within Stated range**	70 % minimum
**Max larger than upper limit**	15 %
**Max finer than lower limit**	15 %
**Bulk Density Loose (kg/m^3)**	1310
**Bulk Density Compact (kg/m^3)**	1510
**Moisture Content**	< 0.1 %
**Air Entrainment**	< 1.5 %
**Clay**	-
**Silt**	-
**Organic Matter**	-

S3. Moisture Retention and Void Ratios of biopolymer stabilised samples after 7 days curing at 20 degrees. A) Moisture Retention (%) of CMC (1.44%, $Mass_{biopolymer}/Mass_{soil}$) stabilised SiO_2 samples upon an increase in M_w ($gmol^{-1}$). B) Void Ratio of CMC (1.44%, $Mass_{biopolymer}/Mass_{soil}$) stabilised samples upon an increase in M_w ($gmol^{-1}$). C) Moisture Retention (%) of GM (G:M 1:X) stabilised SiO_2 and SiO_2 + Fe soil systems. D) Void Ratio of GM (G:M 1:X) stabilised SiO_2 and SiO_2 + Fe soil systems. CMC was utilized as a positive control due to its previously identified lack of Fe binding.

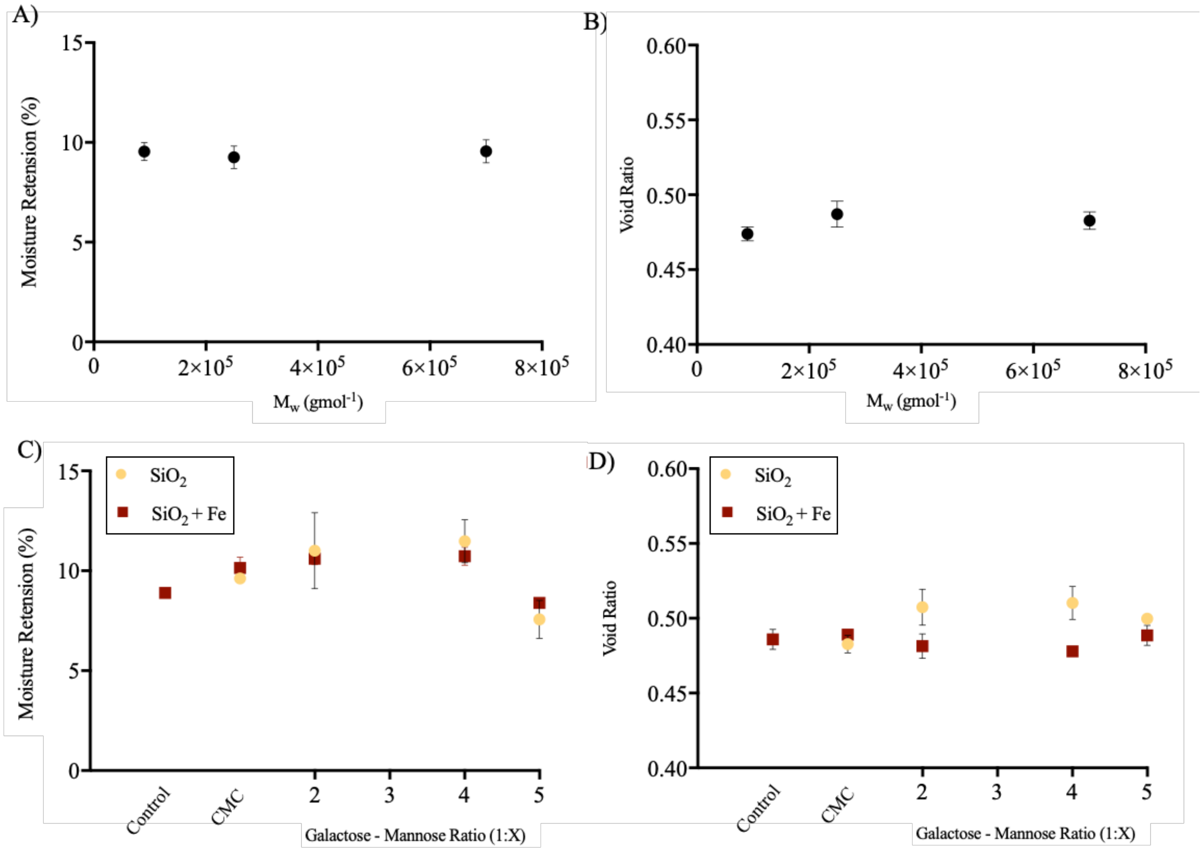

S4. Relationship between UCS (kPa) and Moisture Retention (%) of GM (1%, $Mass_{biopolymer}/Mass_{soil}$) stabilised SiO_2 and SiO_2 + Fe soil systems after 7 days of curing at 20°C. Linear regression statistical analysis, was carried out on each sample set, with the resultant R^2 and *p* – values represented.

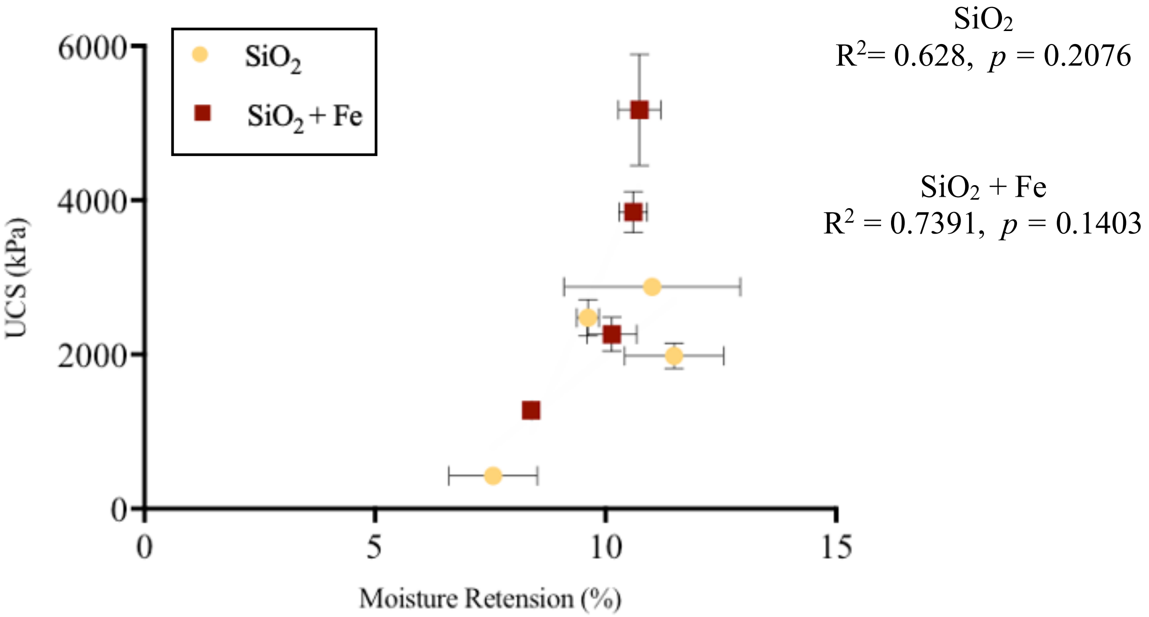

S5. Strength characteristics of CMC (1.44 %, $Mass_{biopolymer}/Mass_{soil}$) stabilised SiO_2 samples upon increasing molecular weight M_w ($gmol^{-1}$), after 7 days curing at 20℃. A) Axial Strain at Peak Strength (%) B) Stiffness Secant at Failure (kN/m).

A)

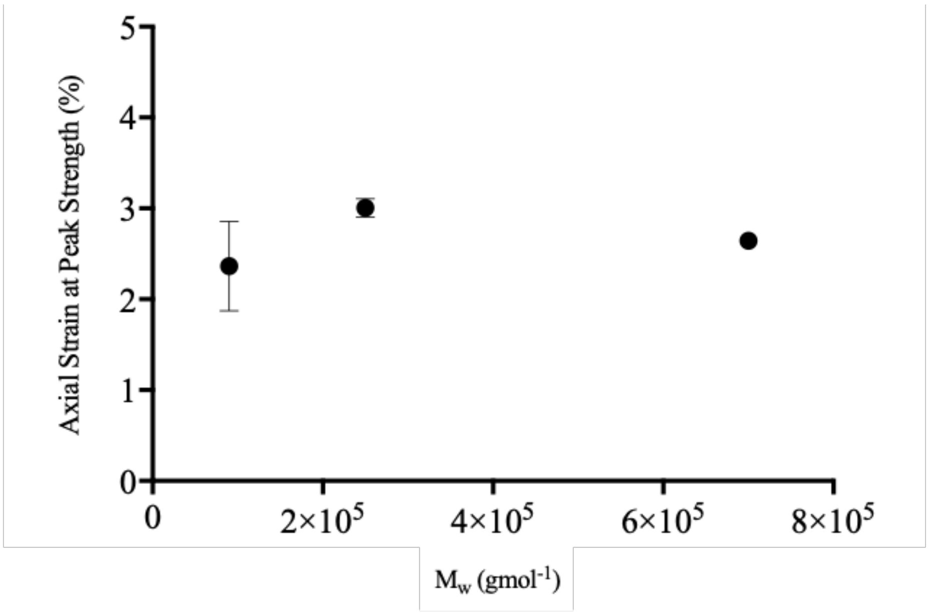

B)

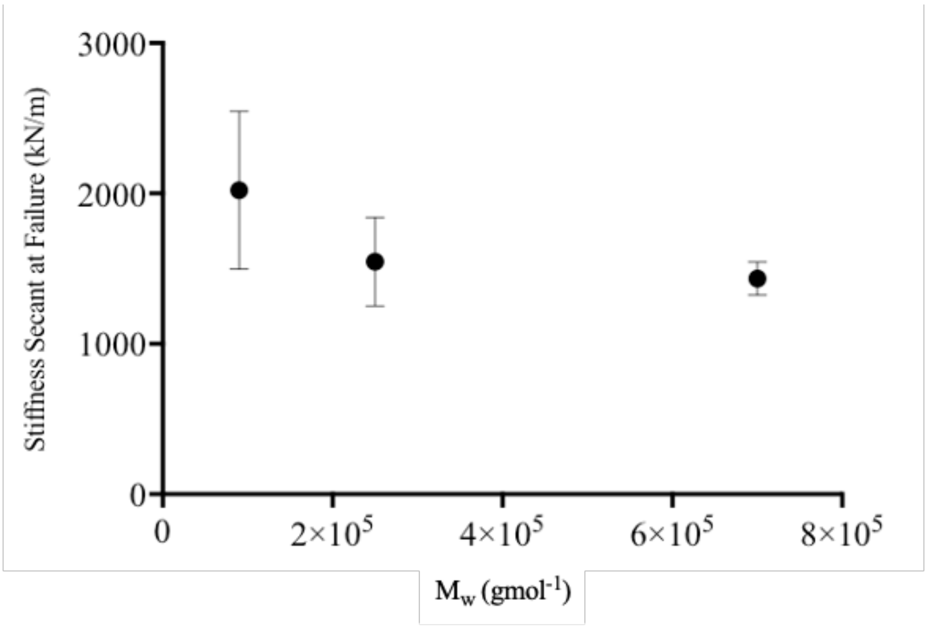

References

1. Li X, Yu H, He Y, Xue X (2012) Synthesis of Fe-MCM-41 Using Iron Ore Tailings as the Silicon and Iron Source. J Anal Methods Chem 1–5
2. Tang C, Li K, Ni W, Fan D (2019) Recovering Iron from Iron Ore Tailings and Preparing Concrete Composite Admixtures. Minerals 9:232
3. Thobakgale R, Gitari WM, Akinyemi SA (2017) Evaluation of the Geochemical and Minerological Transofrmation at an old Copper Mine Tailings Dump in Musina, Limpop Province, South Africe. University of Venda
4. Gitari MW, Akinyemi SA, Thobakgale R, et al (2018) Physicochemical and mineralogical characterization of Musina mine copper and New Union gold mine tailings: Implications for fabrication of beneficial geopolymeric construction materials. J African Earth Sci 137:218–228
5. R. P. Mapinduzi (2016) Potential for reuse of gold mine tailings as secondary construction materials and Phytoremediation. Int J Environ Sci 7:49–61
